# The Preoperative Management of Oral Contraceptive Pills in Aesthetic Plastic Surgery Practice in Saudi Arabia

**DOI:** 10.7759/cureus.31121

**Published:** 2022-11-05

**Authors:** Fahad Aljindan, Noor H Allababidi, Hatan Mortada, Fahad Alhumaid, Salman A Alzaidi

**Affiliations:** 1 Plastic and Reconstructive Surgery, King Abdullah Medical City, Makkah, SAU; 2 Plastic Surgery, Imam Abdulrahman Bin Faisal University, Dammam, SAU; 3 Plastic and Reconstructive Surgery, King Abdulaziz Medical City, Jeddah, SAU; 4 Department of Plastic Surgery & Burn Unit, King Saud Medical City, Riyadh, SAU; 5 Plastic Surgery, King Saud University Medical City, Riyadh, SAU; 6 Plastic and Reconstructive Surgery, King Faisal Specialist Hospital and Research Centre, Jeddah, SAU

**Keywords:** plastic surgergy, aesthetic surgery, hormone-replacement therapy, deep vein thrombosis, oral contraceptive pills

## Abstract

Introduction: Oral contraceptive pills (OCPs) are the most popular contraception method in Saudi Arabia due to their accessibility and reversibility. However, there is no recommendation to stop OCP medication before or after elective aesthetic surgery. The study involves identifying plastic surgeons' behaviors and current practices in perioperative OCPs management in aesthetic surgery in Saudi Arabia, which is the first of its kind.

Methods: A validated self-administered survey was distributed in February 2022 among all board-certified plastic surgeons in Saudi Arabia via social media. The questionnaire was developed to gather information on the perioperative management of OCPs in aesthetic surgery.

Results: A total of 46 board-certified plastic surgeons participated (overall response rate of 48.4%). Among the participants, 32 surgeons (69.6%) indicated that they instruct their patients to discontinue OCPs preoperatively. More than half of surgeons have instructed their patients to stop OCPs after plastic surgery (52.2%). Based on the three occupational characteristics of the surgeons, we found no significant associations between surgeons’ practice patterns regarding OCP discontinuation preoperatively or postoperatively.

Conclusion: In light of the fact that OCPs are reported to pose a risk for venous thromboembolic events, our survey found that most aesthetic surgeons cease their use both preoperatively and postoperatively. There is a need for a guideline regarding perioperative measures for OCPs.

## Introduction

Venous thromboembolism (VTE) is considered a potentially life-threatening event. Surgery is among several factors that may dispose the patient to VTE [1]. In plastic surgery practice, few publications are found in the literature reporting the incidence of deep vein thrombosis and pulmonary embolism [2-4]. The incidence was less than 0.10% among aesthetic plastic surgery patients, though it increased with combined procedures [2,5] Swanson et al. reviewed 1000 patients who underwent cosmetic surgery and found an incidence of 0.9% of deep venous thrombosis (DVT) using perioperative Doppler ultrasound scan [6]. Among 273 surgeons who performed 9937 facelifts, 3.5% had deep vein thrombosis, 0.14% had a pulmonary embolism, and 0.01% died from deep vein thrombosis [3].

Oral contraceptive pills (OCPs) are known to change blood composition. All combined oral contraceptive formulations were investigated in a systematic review and were associated with an increased risk of venous thrombosis [7,8]. British National Formulary recommends discontinuing combined OCPs 4 to 6 weeks before major surgeries, lower limb surgeries, and when long-term immobilization is expected after surgery [9]. Consequently, patients should not begin taking OCP within two weeks of surgery or after total mobilization [10]. The current practice of OCP management in plastic surgery is unclear due to a lack of literature regarding VTE prophylaxis [11]. A survey that included 151 plastic surgeon members of the British Association of Plastic Surgery showed that only 54% of surgeons considered discontinuing combined OCP, and only 20% considered stopping hormone replacement therapy (HRT) [4]. In another study, 1844 surgeons affiliated with the American Society of Plastic Surgery were surveyed. A total of 220 surgeons responded. In fewer than one-third of cases, surgeons discontinued OCPs either preoperatively or postoperatively [12]. On the other hand, a study conducted by Alshardan et al. found that OCPs are one of the most preferred methods of contraception in Saudi Arabia because of their reversibility and accessibility [13]. No guidelines have been published for stopping OCP therapy before or after cosmetic surgery in Saudi Arabia, and no data has been published regarding the current practice of plastic surgeons in Saudi Arabia. This is the first study that aimed to identify current practices and plastic surgeons' behaviors regarding perioperative OCP management in aesthetic surgery in Saudi Arabia.

## Materials and methods

In February 2022, we conducted a survey-based cross-sectional study to evaluate the current practice of plastic surgeons in Saudi Arabia regarding the preoperative management of oral contraceptives.

The ethical committee in King Abdulaziz Medical City, Makkah, Saudi Arabia, approved the study (IRB number: 22-912) With the authors' permission, we developed a validated self-administered questionnaire based on a previous publication [4,10,12]. Prior to distribution, the questionnaire was reviewed by two senior plastic surgery consultants to ensure its ease of completion.

After the feedback was obtained, the questionnaire was distributed among ten plastic surgery residents as a pilot group to ensure the questionnaire was error-free and ready for distribution. The survey was also tested and found to be comprehensible, relevant, and responsive. Saudi Arabian plastic surgeons who are board-certified were asked to complete the questionnaire via social media. A link was shared with Saudi plastic surgeons' WhatsApp groups. In addition, the investigators personally shared the link with plastic surgeons on their contact lists. Board-certified plastic surgeons in active practice were the only criteria for inclusion. Non-board-certified surgeons, residents, and registrars were excluded. This questionnaire was designed to collect information about aesthetic surgical procedures and perioperative discontinuation of OCP. This study did not analyze or test for patient clinical outcomes or patient-reported outcomes. Descriptive statistics, including frequencies and percentages, were used to express the categorical data. A multiple-response analysis was used to describe the frequencies of OCP discontinuation and continuation before and after selected types of surgeries.

We used a chi-square test or Fisher's exact test to assess the univariate association between surgeons' occupational characteristics and OCP practice patterns before and after plastic surgeries. A 0.05 p-value with a 95% confidence interval was used to determine the statistical significance. Statistical analysis was carried out using the Statistical Package for the Social Sciences (SPSS) for Windows, version 26.0 (IBM Corp., Armonk, NY).

## Results

Clinical practice characteristics of the responding surgeons

In the present study, we analyzed the responses of 46 plastic surgery consultants and associate consultants (overall response rate of 48.4%). A total of 16 surgeons (34.8%) were working in a governmental primary practice institution exclusively (university hospitals, national guard affiliated hospitals, military hospitals, etc.), nine surgeons (19.6%) in a private institution, and 21 surgeons (45.7%) in both governmental and private institutions. The majority of surgeons (60.9%) were regularly performing 100 to <300 surgical procedures per year, while 19.6% of them were performing 300 to 500 surgeries per year (Table 1).

**Table 1:** Surgeons’ responses regarding their practice patterns of routine oral contraceptive pills (OCPs) discontinuation before plastic surgery operations.

Parameter	Category	Frequency	Percentage
Routine OCP discontinuation before operations	No	14	30.4
	Yes	32	69.6
Duration of OCP discontinuation before plastic surgeries (n=32)	2 weeks	3	9.4
	4 weeks	21	65.6
	6 weeks	5	15.6
	8 weeks	1	3.1
	12 weeks	2	6.3

**Figure 1:**
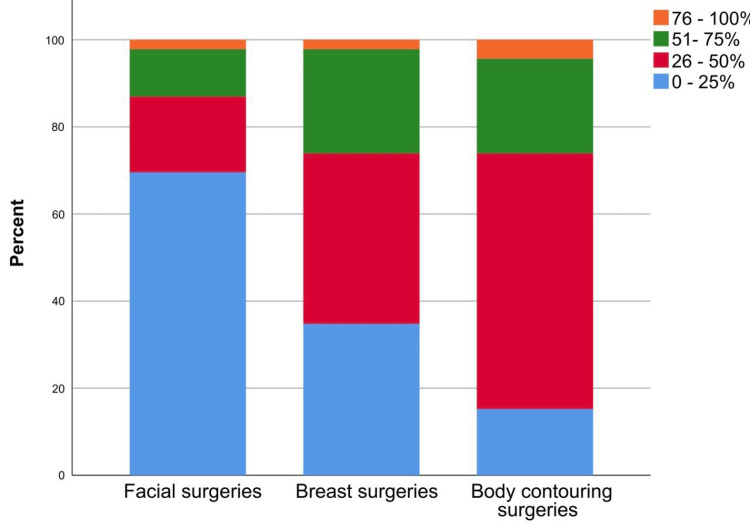
Participants' responses regarding the percentage distribution of plastic surgeries performed by the surgeons.

When the surgeons were asked about the frequency distribution of surgery types, breast surgeries were the most commonly performed procedures, where 26.1% of the respondents declared that breast surgeries represented 51% to 100% of all the surgeries (Figure 1). 

Surgeons' practice regarding OCP cessation before plastic surgeries

In general, 32 surgeons (69.6%) indicated that they instruct their patients to discontinue OCPs preoperatively. Notably, OCPs were stopped four weeks before surgeries by the majority of respondents (65.6%) (Table 2).

**Table 2:** Clinical practice characteristics of the responding surgeons (n=46).

Parameter	Category	Frequency	Percentage
Years in practice	0-5 years	7	15.2
	5- 10 years	10	21.7
	10-15 years	8	17.4
	15-20 years	5	10.9
	> 20 years	16	34.8
Primary practice setting	Governmental only	16	34.8
	Private practice only	9	19.6
	Both Government and Private	21	45.7
Approximate number of procedures performed per year	<100	4	8.7
	100-300	28	60.9
	300-500	9	19.6
	>500	4	8.7
	Missing	1	2.2

According to surgeons' preferences analysis, OCP discontinuation was most common before body contouring surgery (50%) and before breast plastic surgery (35.9%). 

Surgeons' practice regarding OCP cessation after plastic surgeries

**Figure 2:**
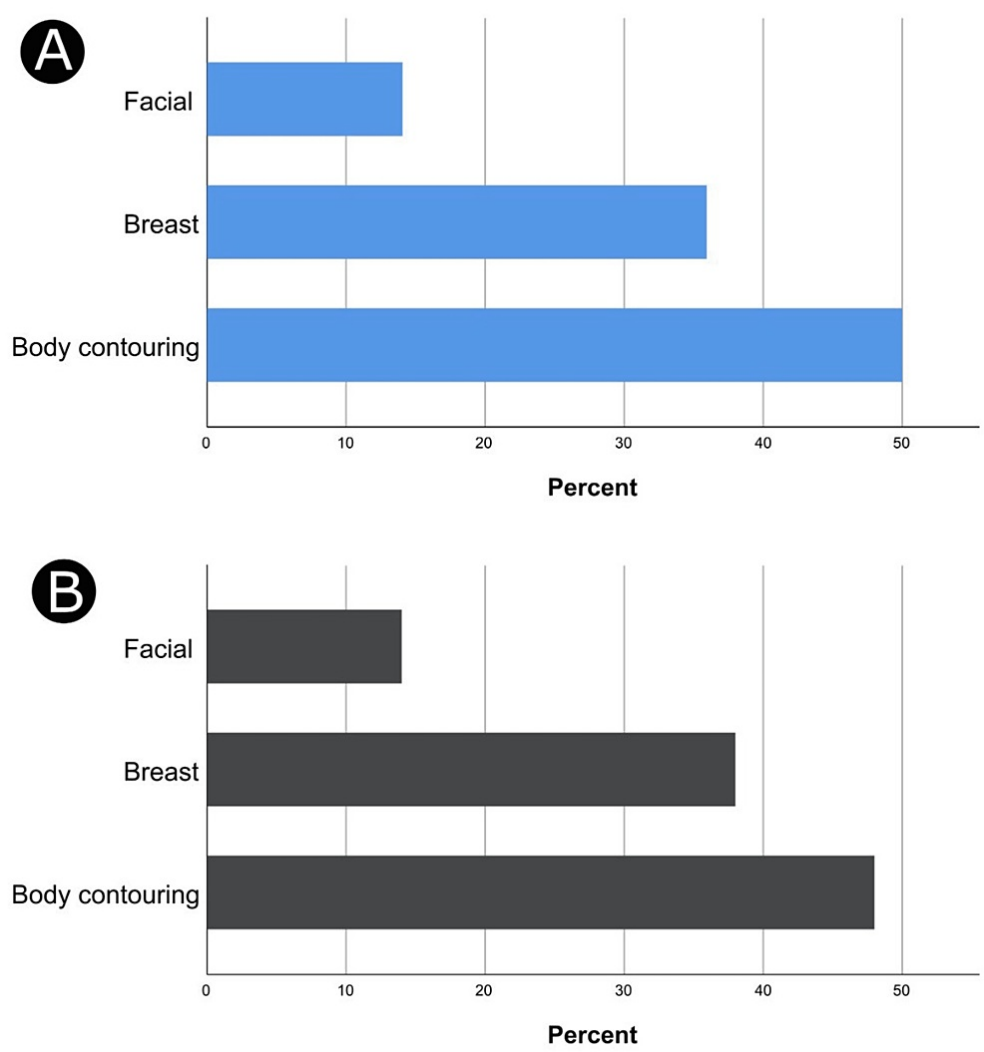
Surgeons’ responses regarding their practice patterns of oral contraceptive pills (OCPs) discontinuation before selected types of plastic surgeries (A) and OCP continuation after those surgeries (B). Results are expressed as percentages, and the results were based on 64 and 50 responses in panels A and B, respectively.

More than half of surgeons have instructed their patients to stop OCPs after plastic surgeries (52.2%). Among these surgeons, OCPs were resumed four weeks after surgeries as indicated by 54.2% of the surgeons (Table 3). Focusing on the types of plastic procedures, surgeons selected 50 multiple selections, among which OCPs were commonly resumed after body contouring surgeries (48.0%) and breast plastic operations (38.0%) (Figure 2).

**Table 3:** Surgeons’ responses regarding their practice patterns of routine oral contraceptive pills (OCPs) continuation after plastic surgery operations.

Parameter	Category	Frequency	Percentage
Routine OCP continuation after operations	No	22	47.8
	Yes	24	52.2
Duration of OCP continuation after plastic surgeries (n=24)	1 week	2	8.3
	2 weeks	5	20.8
	4 weeks	13	54.2
	6 weeks	2	8.3
	12 weeks	2	8.3

Factors associated with OCP instructions before and after plastic surgeries

Based on the three occupational characteristics of the surgeons, we found no significant associations between surgeons’ practice patterns regarding OCP discontinuation before plastic surgeries. Furthermore, there were no significantly associated factors with OCP continuation postoperatively (Table 4).

**Table 4:** Factors associated with oral contraceptive pills (OCPs) discontinuation before and after plastic surgeries.

Parameter	Category	OCP discontinuation before operations				OCP continuation after operations		
		No	Yes	p	No		Yes	p
Years in practice	0-5 years	1 (7.1)	6 (18.8)	0.329	3 (13.6)		4 (16.7)	0.6
	5- 10 years	3 (21.4)	7 (21.9)		6 (27.3)		4 (16.7)	
	10-15 years	1 (7.1)	7 (21.9)		2 (9.1)		6 (25.0)	
	15-20 years	1 (7.1)	4 (12.5)		2 (9.1)		3 (12.5)	
	> 20 years	8 (57.1)	8 (25.0)		9 (40.9)		7 (29.2)	
Primary practice setting	Government Only	6 (42.9)	10 (31.3)	0.705	8 (36.4)		8 (33.3)	0.21
	Private Only	2 (14.3)	7 (21.9)		2 (9.1)		7 (29.2)	
	Both	6 (42.9)	15 (46.9)		12 (54.5)		9 (37.5)	
Approximate number of procedures performed per year	<100	1 (7.7)	3 (9.4)	0.653	1 (4.8)		3 (12.5)	0.692
	100-300	10 (76.9)	18 (56.3)		13 (61.9)		15 (62.5)	
	300-500	1 (7.7)	8 (25.0)		4 (19.0)		5 (20.8)	

## Discussion

Virchow's triad defines the factors associated with the thromboembolism phenomenon [4]. Undergoing surgery may promote factors that might increase the risk of VTE [11]. Evidence reporting the incidence of VTE in plastic surgery patients is lacking [2-4]. Caprini Risk Assessment Model for VTE includes OCPs as a risk factor in stratifying VTE risk [4]. The risk of postoperative DVT increases by two-fold in nonusers (0.5%) compared to users of the pill (1%) [7,8]. There is insufficient evidence linking DVT to hormone supplementation and OCP in plastic surgery patients. A Cochrane review and an article from 2021 indicate that OCP types and doses are associated with DVT risk [7,8]. Despite this, a study conducted by Swanson found that Caprini scores are less valid because they do not correlate with relative risk values [14]. Body contouring procedures post-massive weight loss put the patients at increased risk for VTE due to elevated BMI, extended operative time, concomitant liposuction, and resection weight of over 1500 g. All are considered independent risk factors for VTE in body contouring surgery [11]. Swanson reported that abdominoplasty, long operative times, and the number of procedures were significantly associated with VTE and HRT, however, they did not significantly affect VTE risk [6]. Combining multiple operations in plastic surgery will add more risk to deep vein thrombosis and pulmonary embolism [3,6].

The hemostatic alterations after combined OCP treatment have been demonstrated to be reversed within at least four weeks, lowering the risk of a thromboembolic incident to that of a nonuser [15]. Consequently, combined oral contraceptives should be stopped four weeks before elective surgeries involving a significant risk of deep vein thrombosis and longer surgeries [16]. This is explained by the fact that estrogen changes the ratio of coagulation factors, which increases the risk in a dose-dependent pattern. In our study, most surgeons (65.6%) asked their patients to stop OCPs four weeks before surgery, aligning with the literature; 30.4% of the surgeons did not routinely stop OCPs before operations.

On the other hand, discontinuing combined oral contraceptives may result in an unexpected pregnancy with surgical and anesthetic risks and eventual termination [17,18]. As it is widely known that pregnancy increases the chance of DVT development, with reported figures ranging from 30 to 100 per 100,000 [18,19]. As a result, each patient is treated individually. Because pregnancy increases the chance of DVT, the dangers of discontinuing the combined oral contraceptive must be weighed against the likelihood of an unexpected pregnancy [18,20]. A cross-sectional survey conducted among 285 plastic surgeons by Johnson et al. showed that 54% discontinued OCPs before surgery. In this study, the majority discontinued HRT use for 5-6 weeks before surgery and until full ambulation after surgery [4]. In another study by Chattha et al., only 31.8% of surgeons reported discontinuation of OCPs pre-or postoperatively [12].

Regarding alternatives, shifting the patient to a progesterone-only tablet or depo injection four weeks before surgery are common options for quitting the combined oral contraceptive (progesterone poses no increased risk) [15,18]. Thromboprophylaxis, such as subcutaneous heparin, graded elastic stockings, intermittent pneumatic calf compression, and the preservation of calf muscle pump by total intravenous anesthesia without paralysis must be explored to decrease the risk of VTE [16,21,22]. It is necessary to think about the process that will be carried out. Although elective cosmetic surgery lasts more than an hour in people over the age of 40 years old, it is associated with a modest risk of thrombosis. Before major surgery, most plastic surgeons surveyed stopped using combined oral contraceptives. Regardless of the type of procedure, all additional variables that predispose to DVT must be examined [16]

In our study, surgeons' preference analysis showed that OCP discontinuation was standard before body contouring surgeries (50%) and breast plastic operations (35.9%). We found no significant associations between surgeons' practice type and OCP discontinuation before plastic surgery. Despite some recommendations, there is no sufficient evidence to support stopping HRT before surgery [16,17]. The hazards of withdrawal, such as low mood, frequent daytime flushes, and night sweats, are avoided by maintaining HRT [17,18,23].

Based on our findings, in the future, we recommend that high-quality study designs, such as randomized control trials, be used to investigate discontinuing OCPs before making any reliable recommendations. In addition, we believe that future studies are needed to address the effect of oral contraceptives on bleeding, edema, inflammation, and scarring postoperatively. The study included a large and diverse sample. In addition, a significant strength of the current study is that we used a previously validated questionnaire to evaluate the current practices regarding perioperative OCP management in aesthetic surgery in Saudi Arabia. According to a systematic review by Santesso et al., studies using validated questionnaires can make reliable conclusions [24]. All future studies should use validated questionnaires to come up with trustworthy conclusions, as we recommend in future studies. The validity of a survey can be categorized into three different types: construct validity, convergent validity, and content validity. By determining what survey questions to use, validity ensures that researchers are using questions that measure the issues that matter. Validity is determined by whether a survey measures what it claims to measure. Valid survey results require the use of high-quality scientific research [24]. 

Even though the study's objectives have been achieved, several limitations must be mentioned. The first limitation includes a moderate response rate among individuals surveyed. The second limitation is that the study's cross-sectional design might have been subjected to recall bias, question misinterpretation, and reporter bias. The last limitation is the failure to determine the type of anesthesia used. For example, a plastic surgeon using sedation and a local anesthetic for a facelift or liposuction would not need to employ deep vein thrombosis prophylaxis and might not discontinue the use of OCPs. It is also worth mentioning that HRT, being an artificial estrogen, might also be regarded as raising DVT risk, even though there is little evidence to back this up. More research is needed to evaluate the danger, but until then, HRT should be treated the same way as combined oral contraceptives. It is hoped that guidelines regarding oral contraceptives and aesthetic surgery will be published in Saudi Arabia in the near future.

## Conclusions

This cross-sectional study design focused only on providers’ practices of preoperative management of OCPs. We recommend that plastic surgeons evaluate the intake of OCPs prior to surgery and that patients understand the risks and benefits of using OCPs in the perioperative period. Additionally, surgeons should advise patients to use alternative contraception methods. Further studies should include a comprehensive algorithm to guide the use of OCP in plastic surgery and examine the relation of OCP to edema, scarring, and bleeding.

## References

[1] McLendon K, Goyal A, Attia M (2022). Deep Venous Thrombosis Risk Factors. https://www.ncbi.nlm.nih.gov/books/NBK470215/.

[2] Winocour J, Gupta V, Kaoutzanis C, Shi H, Shack RB, Grotting JC, Higdon KK (2017). Venous thromboembolism in the cosmetic patient: analysis of 129,007 patients. Aesthet Surg J.

[3] Reinisch JF, Bresnick SD, Walker JW, Rosso RF (2001). Deep venous thrombosis and pulmonary embolus after face lift: a study of incidence and prophylaxis. Plast Reconstr Surg.

[4] Johnson RL, Hemington-Gorse SJ, Dhital SK (2008). Do cosmetic surgeons consider estrogen-containing drugs to be of significant risk in the development of thromboembolism?. Aesthetic Plast Surg.

[5] White AJ, Kanapathy M, Nikkhah D, Akhavani M (2021). Systematic review of the venous thromboembolism risk assessment models used in aesthetic plastic surgery. JPRAS Open.

[6] Swanson E (2020). Prospective study of Doppler ultrasound surveillance for deep venous thromboses in 1000 plastic surgery outpatients. Plast Reconstr Surg.

[7] de Bastos M, Stegeman BH, Rosendaal FR, Van Hylckama Vlieg A, Helmerhorst FM, Stijnen T, Dekkers OM (2014). Combined oral contraceptives: venous thrombosis. Cochrane Database Syst Rev.

[8] Morimont L, Haguet H, Dogné JM, Gaspard U, Gaspard J (2021). Combined oral contraceptives and venous thromboembolism: review and perspective to mitigate the risk. Front Endocrinol.

[9] (2022). Faculty of Sexual & Reproductive Healthcare (FSRH): Combined hormonal contraception. FSRH.

[10] Broughton G II, Rios JL, Rohrich RJ, Brown SA (2007). Deep venous thrombosis prophylaxis practice and treatment strategies among plastic surgeons: survey results. Plast Reconstr Surg.

[11] Clavijo-Alvarez JA, Pannucci CJ, Oppenheimer AJ, Wilkins EG, Rubin JP (2011). Prevention of venous thromboembolism in body contouring surgery: a national survey of 596 ASPS surgeons. Ann Plast Surg.

[12] Chattha A, Brown E, Slavin S, Lin S (2018). Oral contraceptive management in aesthetic surgery: a survey of current practice trends. Aesthet Surg J.

[13] Alshardan A, Bari M, AlSinan I, AlMuqhim M, AlRazeyg N (2020). Knowledge and use of contraceptives among women in Al-Kharj City, Saudi Arabia. Int J Med Dev Ctries.

[14] Swanson E (2016). Caprini scores, risk stratification, and rivaroxaban in plastic surgery: time to reconsider our strategy. Plast Reconstr Surg Glob Open.

[15] Whitehead EM, Whitehead MI (1991). The pill, HRT and postoperative thromboembolism: cause for concern?. Anaesthesia.

[16] Abou-Ismail MY, Citla Sridhar D, Nayak L (2020). Estrogen and thrombosis: a bench to bedside review. Thromb Res.

[17] Thromboembolic Risk Factors (THRIFT) Consensus Group (1992). Risk of and prophylaxis for venous thromboembolism in hospital patients. BMJ.

[18] (1999). Drugs in the peri-operative period: 3--hormonal contraceptives and hormone replacement therapy. Drug Ther Bull.

[19] Heit JA (2002). Venous thromboembolism epidemiology: implications for prevention and management. Semin Thromb Hemost.

[20] Farrell RJ, Lamb J (1988). Should the pill be stopped preoperatively?. Br Med J (Clin Res Ed).

[21] Swanson E, Gordon RJ (2017). Comparing a propofol infusion with general endotracheal anesthesia in plastic surgery patients. Aesthet Surg J.

[22] Swanson E (2022). A new approach for venous thromboembolism prevention in plastic surgery. Plast Reconstr Surg Glob Open.

[23] Guillebaud J (1985). Surgery and the pill. Br Med J (Clin Res Ed).

[24] Santesso N, Barbara AM, Kamran R, Akkinepally S, Cairney J, Akl EA, Schünemann HJ (2020). Conclusions from surveys may not consider important biases: a systematic survey of surveys. J Clin Epidemiol.

